# Genome-wide identification and expression profile of HD-ZIP genes in physic nut and functional analysis of the *JcHDZ16* gene in transgenic rice

**DOI:** 10.1186/s12870-019-1920-x

**Published:** 2019-07-08

**Authors:** Yuehui Tang, Jian Wang, Xinxin Bao, Mengyu Liang, Huimin Lou, Junwei Zhao, Mengting Sun, Jing Liang, Lisha Jin, Guangling Li, Yahui Qiu, Kun Liu

**Affiliations:** 1 Key Laboratory of Plant Genetics and Molecular Breeding, Zhoukou Normal University, Zhoukou, Henan China; 2 Henan Key Laboratory of Crop Molecular Breeding and Bioreactor, Zhoukou, Henan China; 3 School of Journalism and Communication, Zhoukou Normal University, Zhoukou, Henan China; 4 College of Life Science and Agronomy, Zhoukou Normal University, Zhoukou, Henan China

**Keywords:** HD-ZIP gene family, Physic nut, Expression profile, Abiotic stress, *JcHDZ16*

## Abstract

**Background:**

Homeodomain-leucine zipper (HD-ZIP) transcription factors play important roles in the growth, development and stress responses of plants, including (presumably) physic nut (*Jatropha curcas*), which has high drought and salinity tolerance. However, although physic nut’s genome has been released, there is little knowledge of the functions, expression profiles and evolutionary histories of the species’ HD-ZIP genes.

**Results:**

In this study, 32 *HD-ZIP* genes were identified in the physic nut genome (*JcHDZs*) and divided into four groups (I-IV) based on phylogenetic analysis with homologs from rice, maize and *Arabidopsis*. The analysis also showed that most of the *JcHDZ* genes were closer to members from *Arabidopsis* than to members from rice and maize. Of the 32 *JcHDZ* genes, most showed differential expression patterns among four tissues (root, stem cortex, leaf, and seed). Expression profile analysis based on RNA-seq data indicated that 15 of the *JcHDZ* genes respond to at least one abiotic stressor (drought and/or salinity) in leaves at least at one time point. Transient expression of a JcHDZ16-YFP fusion protein in *Arabidopsis* protoplasts cells showed that JcHDZ16 is localized in the nucleus. In addition, rice seedlings transgenically expressing *JcHDZ16* had lower proline contents and activities of antioxidant enzymes (catalase and superoxide dismutase) together with higher relative electrolyte leakage and malondialdehyde contents under salt stress conditions (indicating higher sensitivity) than wild-type plants. The transgenic seedlings also showed increased sensitivity to exogenous ABA, and increases in the transcriptional abundance of several salt stress-responsive genes were impaired in their responses to salt stress. Further data on *JcHDZ16*-overexpressing plants subjected to salt stress treatment verified the putative role of *JcHDZ* genes in salt stress responses.

**Conclusion:**

Our results may provide foundations for further investigation of functions of *JcHDZ* genes in responses to abiotic stress, and promote application of *JcHDZ* genes in physic nut breeding.

**Electronic supplementary material:**

The online version of this article (10.1186/s12870-019-1920-x) contains supplementary material, which is available to authorized users.

## Background

Abiotic stresses, such as drought and salinity, can have severe (or even lethal) effects on plants’ growth, development, and performance (e.g. in terms of crop yields). Thus, plants have evolved diverse adaptive mechanisms that provide varying degrees of resistance or tolerance, controlled by complex regulatory networks [1]. Important elements of these regulatory networks are transcription factors, proteins that regulate the expression of various target genes by binding to cis-acting regulatory elements in their promoter regions [2]. Many transcription factors, including members of the AP2/ERF, NAC, MYB, bHLH, WRKY and HD-ZIP families, have been identified, characterized and shown to participate in regulation of plants’ responses to abiotic stresses [3–8].

HD-ZIP transcription factors, comprising one of the largest gene families in plants, contain two conserved functional domains: a homeodomain (HD) and a leucine zipper (LZ) motif [9, 10]. The HD is responsible for sequence-specific DNA binding, which regulates downstream genes’ transcription, and the LZ motif (which is tightly linked to the HD) mediates homo- and hetero-dimerization [9, 10]. Other domains are also found in some HD-ZIP proteins, such as the MEKHLA domain, which is probably involved in light signaling, and START domain with putative lipid-binding capability [9]. Based on amino acid sequence similarities, conserved domains and their functions, and structural characteristics, members of the HD-ZIP family in *Arabidopsis* are classified into four groups, designated I-IV [11].

Since the first isolation of an HD-containing gene, *KNOTTED1* from maize [12], numerous HD-ZIP transcription factors have been identified in diverse plant species (e.g., *Arabidopsis*, rice, soybean, wheat, maize, poplar, grape, and cassava) through genome-wide analyses [11, 13–19]. Subsequent studies have indicated that *HD-ZIP* genes are involved in numerous plant developmental processes. For example, *TaHDZipl-2* reportedly participates in regulation of flowering time and spike development in wheat and barley transgenically expressing it [20]. *PtrHB4*, a HD-Zip III gene, functions in regulation of interfascicular cambium development [21]. *Hat3* and *athb4* double mutants exhibit organ polarity defects, while *hat3athb4athb2* triple mutation results in cotyledon formation defects and inhibition of shoot apical meristem activity [22]. In *Arabidopsis*, *ATHB17* is involved in regulating chloroplast number and photosynthetic capacity, and its overexpression induces increases in chlorophyll content [23]. *ATHB4* participates in integration of shade perception and hormone-mediated growth [24]. *ATHB12* acts as a positive regulator of leaf growth by promoting cell expansion and endoreduplication [25]. In rice, *OsHox33* knockdown accelerates leaf senescence by regulating expression of *GS1* and *GS2* [26]. *OsHox32* is reportedly involved in leaf development, and transgenic plants overexpressing it produce narrow leaves [27]. *OsHox4* plays an important role in GA deactivation in rice by controlling expression of DELLA subfamily genes [28]. In addition to the functions described above, numerous studies have shown that *HD-ZIP* genes are involved in regulating plant responses to abiotic stress [29, 30]. For example, overexpression of the class I homeodomain transcription factor *TaHDZipI-5* increases drought and frost tolerance in transgenic wheat [6]. Over-expression of *OsHOX24* imparts higher sensitivity to stress hormone, ABA, and abiotic stresses in the transgenic *Arabidopsis* plants [29]. Transgenic expression of *Zmhdz10* increases rice and *Arabidopsis* plants’ tolerance of salinity and drought stresses [30]. Thus, various genes of the HD-ZIP family have been cloned and functionally studied, but there is little knowledge of its members and their functions in many taxa, including the Euphorbiaceae.

Physic nut (*Jatropha curcas*), a perennial shrub of the Euphorbiaceae, has been extensively planted in tropical and sub-tropical regions due to its high seed oil content, ease of propagation, rapid growth, ability to fix sand, and strong tolerance of drought and salinity [31, 32]. The recent release of its genome has provided opportunities for genome-wide identification, classification and comparative genome studies [33]. However, no information on the identification, classification, expression profiles or functions of *HD-ZIP* genes in physic nut has been previously published. To address this gap, we searched for and identified 32 *HD-ZIP* genes in the physic nut genome (hereafter *JcHDZ* genes). We then analyzed the structure, phylogeny, conserved motifs and chromosomal localizations of these genes. Next, we examined tissue expression profiles of the identified genes, under non-stressed conditions and following exposure to drought and salinity. Finally, we transgenically expressed the *JcHDZ16* gene in rice and characterized its function. The results provide insights into the evolution of *JcHDZ* genes, and foundations for exploring roles of *JcHDZ* genes in responses to drought and salinity stresses. They should also facilitate further research into molecular mechanisms underlying stress responses in physic nut, and plants generally.

## Results

### Identification of HD-ZIP gene family members in physic nut

To identify *JcHDZ* genes in the physic nut genome, we performed BLASTP searches using all known HD-ZIP protein sequences from *Arabidopsis* and rice. In addition, the HMM *HD-Zip* gene model was used to detect *JcHDZ* genes that may have been missed. In total, 32 *JcHDZ* genes were finally identified in physic nut, with confirmed presence of both LZ and HD domains according to PFam and SMART database searches. These genes were provisionally designated *JcHDZ01* to *JcHDZ32* based on their positions from top to bottom in the physic nut chromosomes or, more strictly, Linkage Groups (LGs) 1 to 11. The length of the *JcHDZ* genes’ ORFs varied from 549 bp (*JcHDZ01*) to 2541 bp (*JcHDZ17*), thus the encoded proteins potentially range from 182 to 846 amino acids, and their GenBank accession numbers are listed in Additional file 1. Predicted molecular weights and theoretical pI values (isoelectric points) of the 32 deduced JcHDZ proteins range from 21.2 to 92.7 KDa, and 4.66 to 8.95, respectively (Additional file 1).

### Phylogenetic analysis of HD-ZIP gene family

**Fig. 1:**
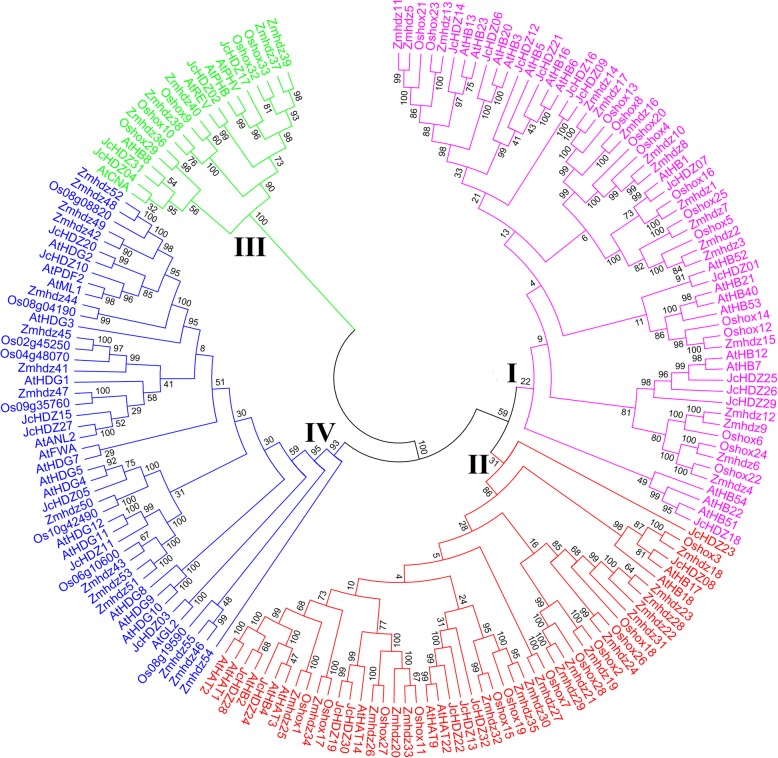
Unrooted phylogenetic tree of HD-ZIP proteins from physic nut, *Arabidopsis*, rice and maize constructed by the neighbor-joining method using MEGA6. Numbers on the nodes indicate clade credibility values

To study the phylogenetic relationships of the 32 HD-ZIP transcription factors in physic nut with previously reported members in other plants, we constructed an unrooted phylogenetic tree using the neighbor-joining method, implemented in MEGA6 software, according to full-length amino acid sequence similarities and topologies. The other HD-ZIP proteins were 44 and 55 from the monocots rice and maize, respectively, and 48 from the dicot *Arabidopsis* [11, 15, 16]. As shown in the resulting phylogenetic tree (Fig. 1), the 179 HD-ZIP proteins were divided into four groups, designated I-IV based on the previous classification of members in rice, maize and *Arabidopsis* [11, 15, 16]. Of the 32 inferred physic nut HD-ZIP proteins, 12 were assigned to group I (JcHDZ01, 06, 07, 09, 12, 14, 16, 18, 21, 25, 26 and 29), 9 to group II (JcHDZ08, 13, 19, 22, 23, 24, 28, 30 and 32), four to group III (JcHDZ02, 04, 17 and 31) and seven to group IV (JcHDZ03, 05, 10, 11, 15, 20 and 27) (Fig. 1). The tree also suggested that most of the JcHDZ proteins were closer to *Arabidopsis* members than to members from rice and maize, as shown in Fig. 1. For example, JcHDZ17 and JcHDZ02 clustered with AtPHV, AtPHB and AtREV in group III, whereas Zmhdz39, Zmhdz37, Oshox32, and Oshox33 from group III in a separate clade.

### Analysis of *JcHDZ* genes’ structure and conserved motifs

**Fig. 2:**
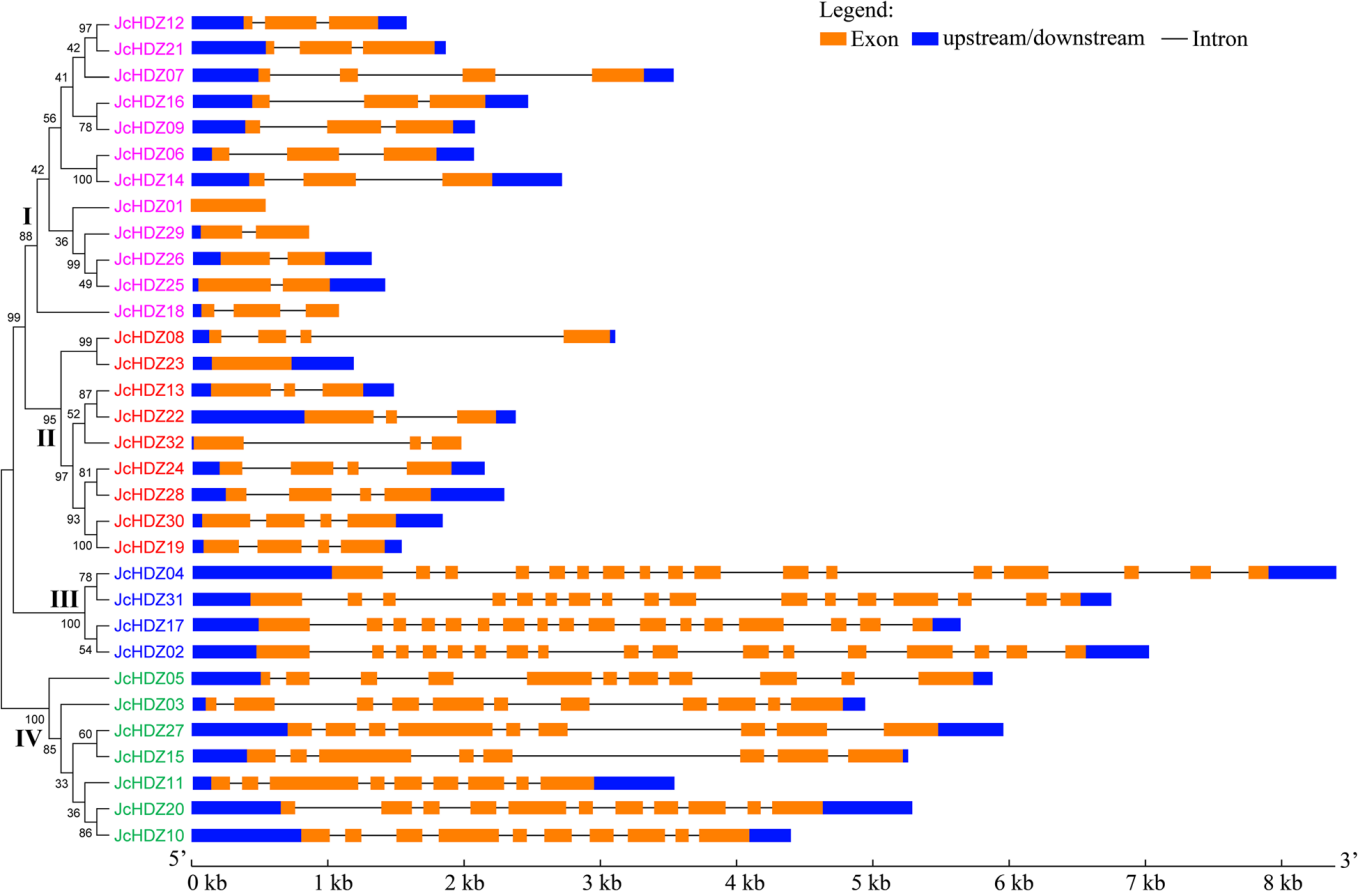
Phylogenetic relationship among the JcHDZ proteins and exon-intron structures of *JcHDZ* genes. Exons and introns are shown as orange boxes and thin lines respectively. Untranslated region (upstream/downstream) are shown as blue boxes. The unrooted tree was constructed, using the MEGA6.0 program, by the neighbor-joining method. Gene classes are indicated with different colors

Analysis of genes’ exon/intron structural characteristics can provide important information on the evolution and phylogeny of gene families [15, 16]. Thus, to evaluate the evolutionary history and diversity of the identified physic nut *JcHDZ* genes, we analyzed their exon/intron structures by comparing their coding sequences (CDS) with the corresponding genome sequences on the GSDS website. The results indicate that all the *JcHDZ* genes have at least one intron, except *JcHDZ01* and *JcHDZ23* (Fig. 2), generally with the very highly conserved splicing arrangements previously reported for *Arabidopsis*, rice, and maize homologs [11, 15, 16]. Most of the genes that clustered in the same group generally have a similar exon/intron structure, especially in terms of intron numbers and exon lengths. For example, all members of group III appear to have 16 introns, whereas all those in group II except *JcHDZ23* have 2–3 introns (Fig. 2). Furthermore, we found that *JcHDZ* genes of group III have more highly conserved exon numbers than members of the other groups (Fig. 2). The highly conserved exon/intron structure of *JcHDZ* genes within each group support the classification of these genes in the phylogenetic tree.

**Fig. 3:**
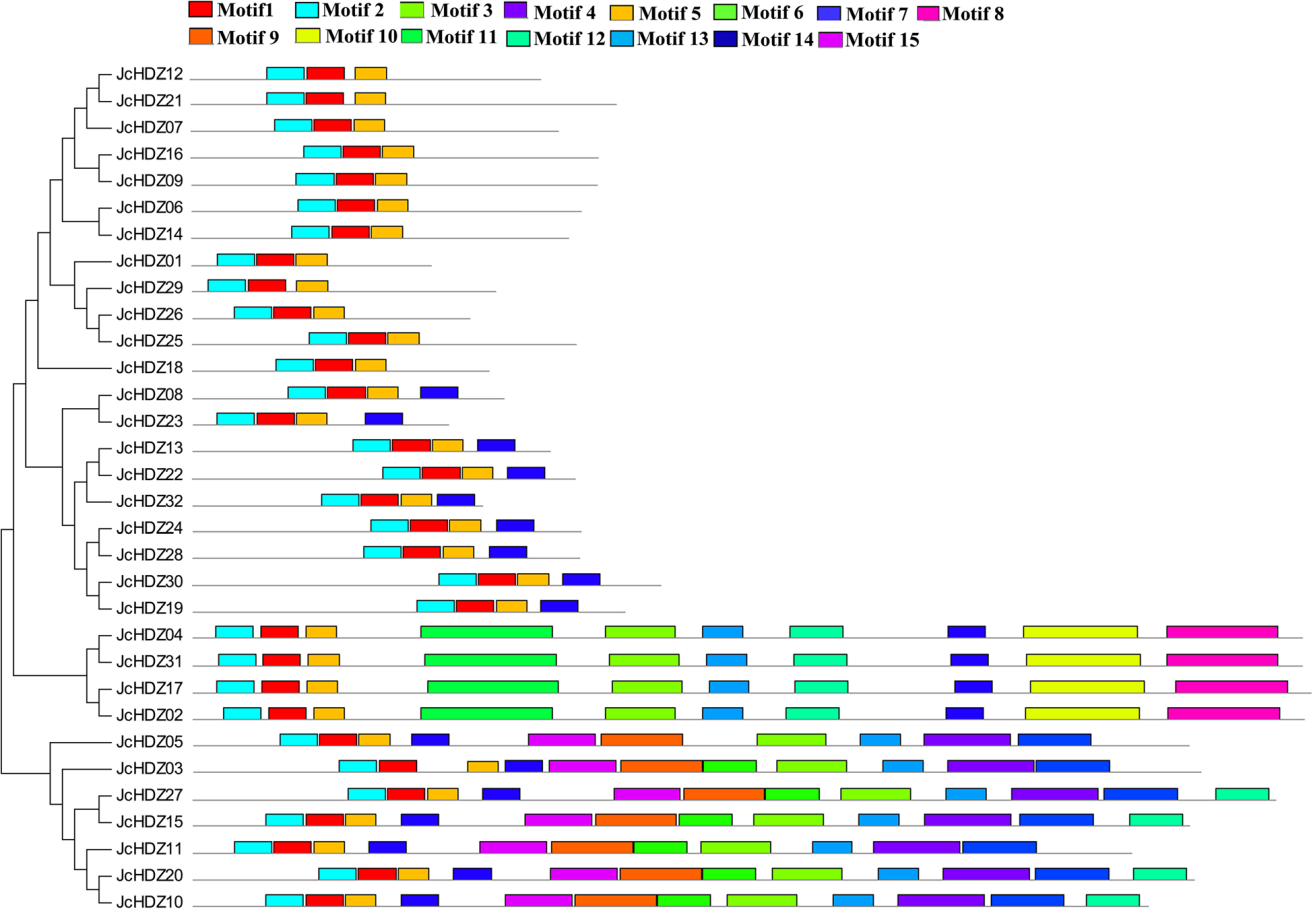
Conserved motifs in JcHDZ proteins. Motifs were determined using MEME suite version 4.12. Grey lines represent non-conserved sequences, and motifs are indicated by colored boxes numbered at the top

We then further analyzed conserved motifs of the putative JcHDZ proteins using MEME software, which detected 15 motifs, in total, in the 32 JcHDZ proteins, which were designated 1 to 15 (Fig. 3 and Additional file 2). As expected, these included an LZ domain (motif 5) and HD domain (motifs 1 and 2) in all identified JcHDZ proteins. A START domain (motif 3) was found in members of groups III and IV, but not groups I and II (Fig. 3). A MEKHLA domain, corresponding to motif 8, was only detected in members of group III. Besides these known functional motifs, some with unknown functions were found. Examples include motifs 4, 7, 9, 11 and 15 (detected only in members of group IV), and motif 10 (found only in members of group III). Motif 14 was found in JcHDZ proteins of groups II, III and IV (Fig. 3). The results also indicate that members of the same *JcHDZ* group generally have similar motifs, and thus might have functional similarities.

### Chromosomal localization analysis of *JcHDZ* genes

**Fig. 4:**
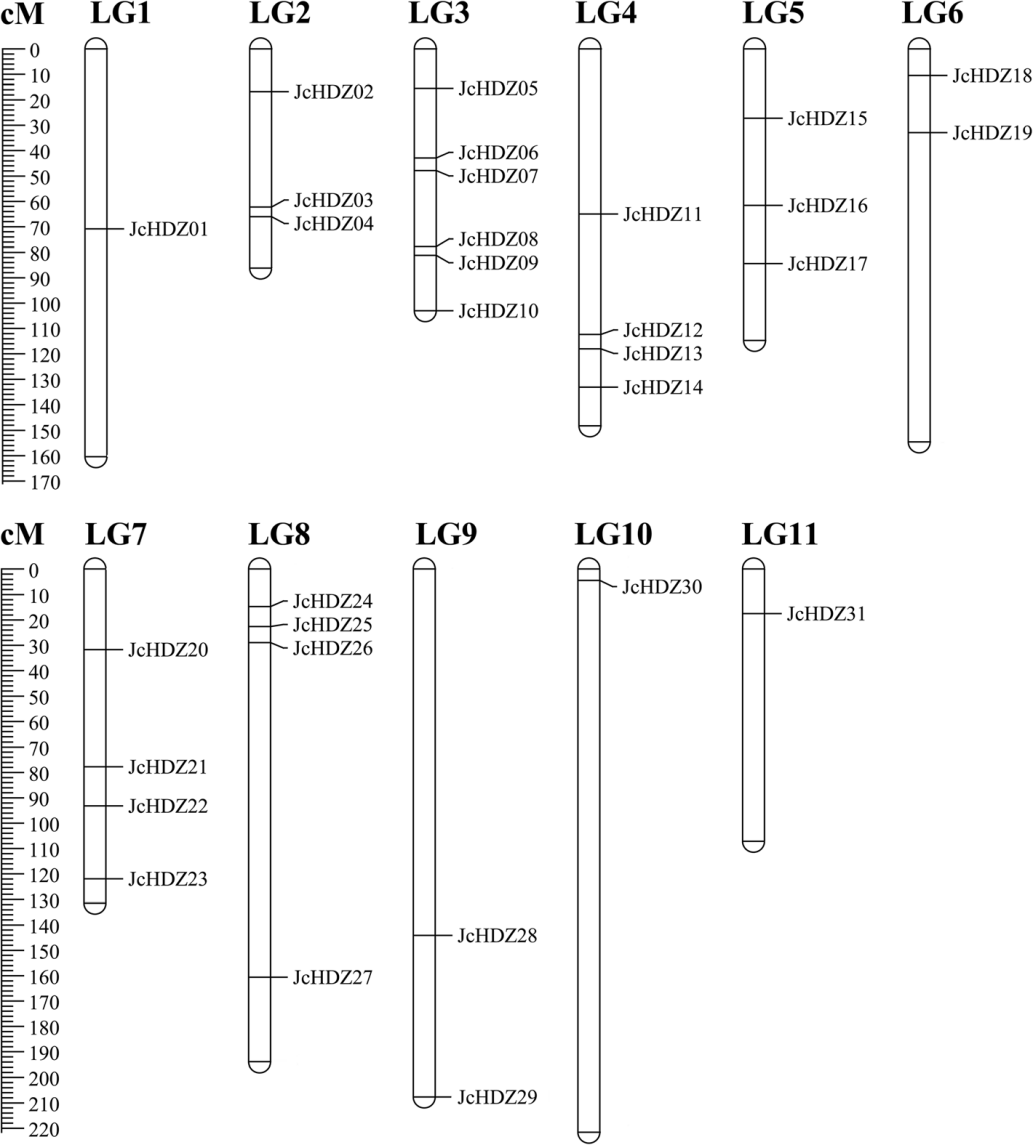
Distribution of *JcHDZ* genes on physic nut chromosomes according to the linkage map. In total, 31 *JcHDZ* genes were mapped to nine linkage groups (LGs). The scale is in centiMorgans (cM)

We mapped 31 of the 32 *JcHDZ* genes (all except *JcHDZ32*) mapped to LGs using previously published information [33]. As shown in Fig. 4, we found that numbers of these genes in specific LGs range from one (in LGs 1, 10 and 11) to six (in LG 3). Twenty of the genes appear to be located in middle or lower positions of LGs, and 12 in upper positions (Fig. 4). We detected no tandem duplications, defined as tandem repeats separated by < 4 non-homologous spacer or located within 50 kb of each other [34] in any identified *JcHDZ* genes.

### Expression profile analysis of *JcHDZ* genes under normal growth conditions

**Fig. 5:**
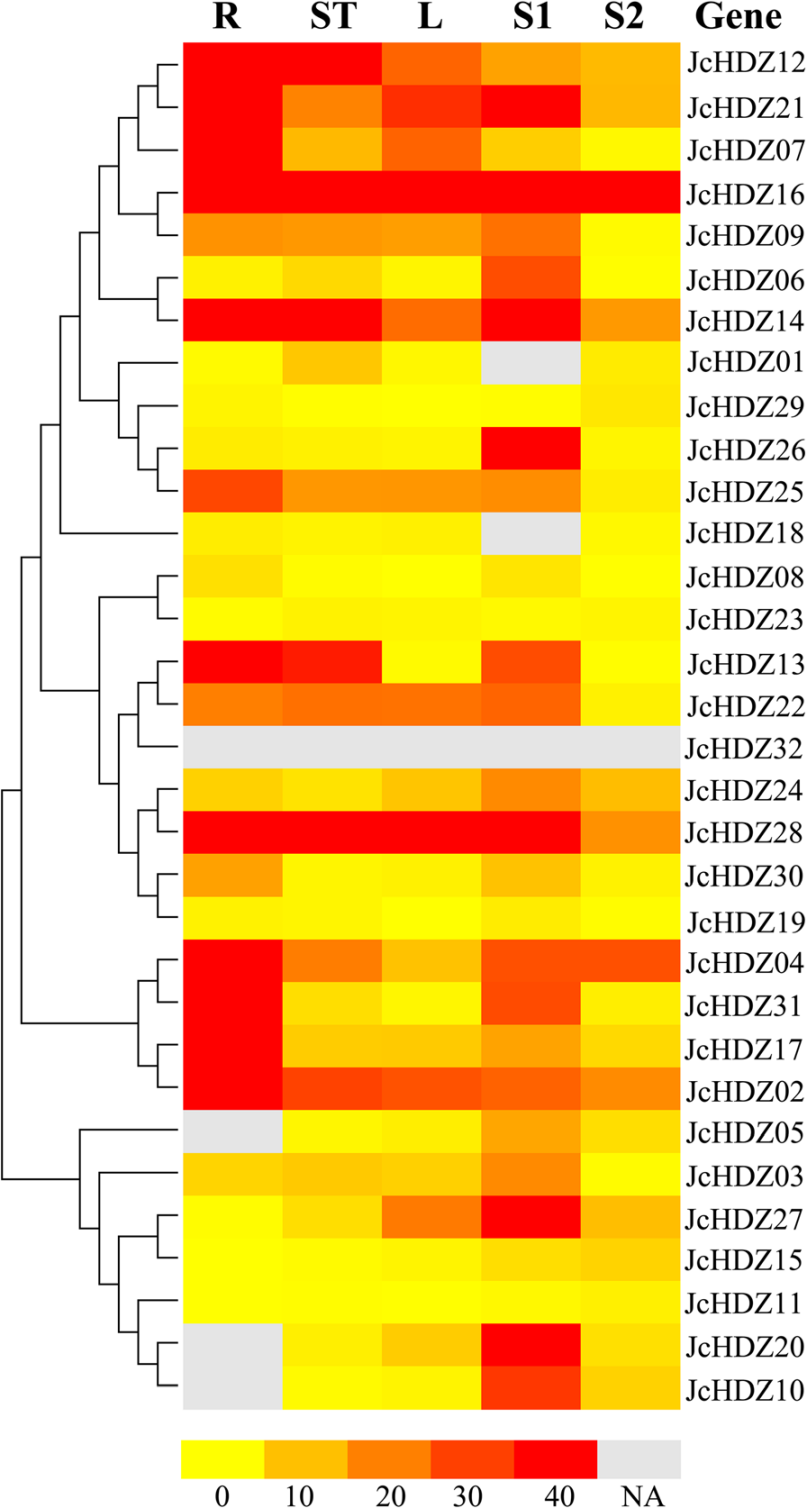
Relative expression levels of each *JcHDZ* gene in physic nut roots (R), stem cortex (ST), leaves (L), and seeds in both an early development stage (S1) and filling stage (S2), with a colored scale of expression levels shown at the bottom. NA: not available

To characterize expression profiles of the *JcHDZ* genes in physic nut under non-stressed growth conditions, we analyzed the abundance of their transcripts in roots, stem cortex, leaves, and seeds (S1 and S2) based on RNA sequencing (RNA-seq) data (Fig. 5 and Additional file 3). Our results suggest that 28 of the detected *JcHDZ* genes are expressed differentially in all sampled organs, three (*JcHDZ05*, *10* and *20*) are not expressed in roots, and we detected no expression of the other gene (*JcHDZ32*) in any sampled organ. Of the 31 *JcHDZ* genes with detected expression, five (*JcHDZ06*, *10*, *20*, *26* and *27*) were most strongly expressed in seeds, and five (*JcHDZ02*, *04*, *17*, *25* and *31*) more strongly in roots than in the other tested organs. In addition, two (*JcHDZ14* and *16*) showed constitutive expression, with high expression levels in all tested organs.

In addition, as shown in Fig. 5, most of the *JcHDZ* genes were more highly expressed in seeds in the S1 stage (14 days after pollination, DAP) than in the S2 stage (41 DAP). However, expression of *JcHDZ01* and *18* was detected in seeds in the S2 stage, but not the S1 stage.

### Expression profile analysis of *JcHDZ* genes under abiotic stress conditions

**Fig. 6:**
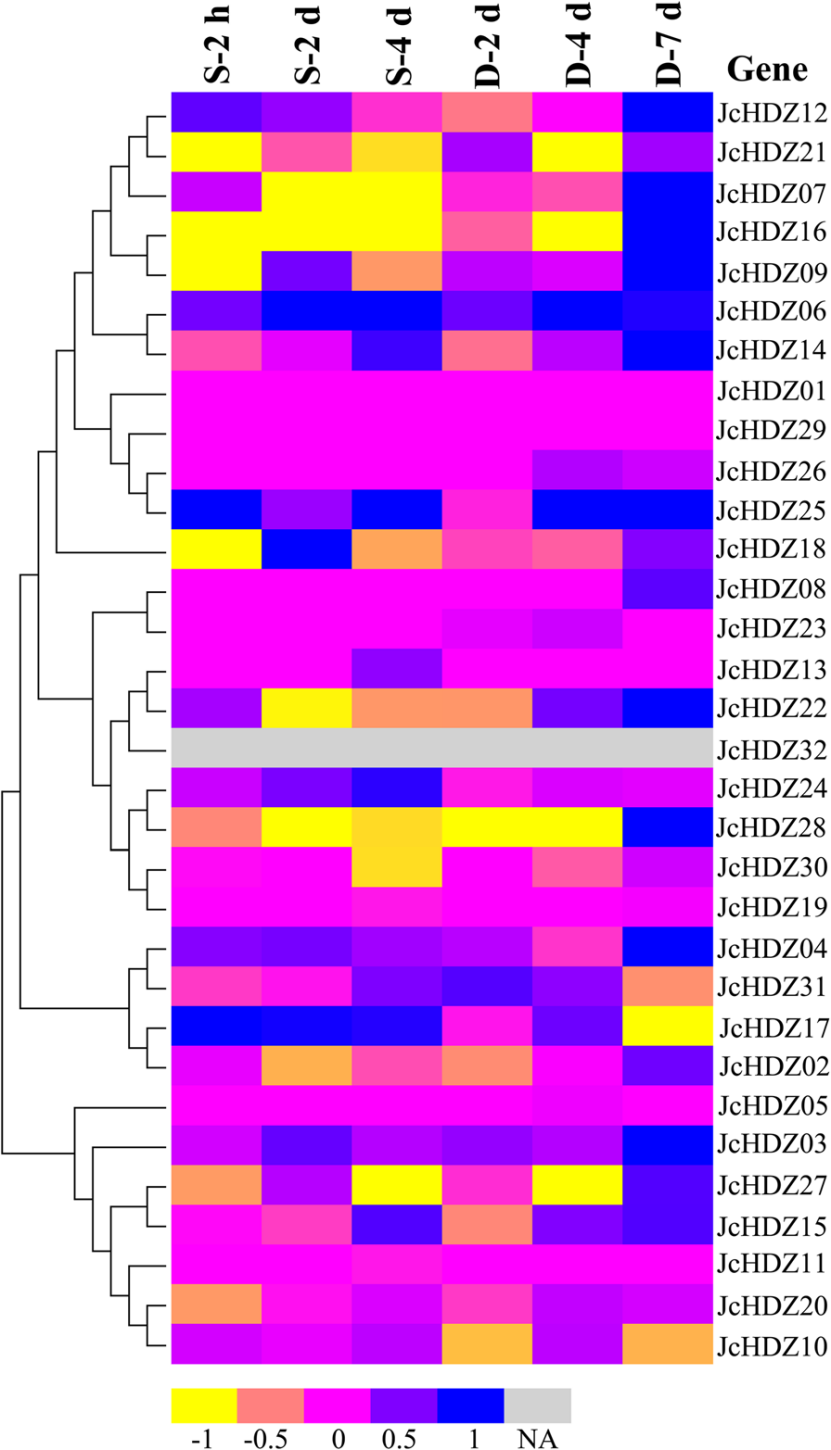
Expression levels of the 32 *JcHDZ* genes in physic nut leaves under drought and salinity stresses: log_2_ ratios of signals from treated versus control leaves in a heat map based on transcriptomic data, with color scale shown at the bottom. NA: not available

Numerous studies have recently demonstrated that HD-ZIP genes are involved in regulation of plants’ responses to abiotic stresses such as drought and salinity [29, 30]. Thus, we examined the abundance of *JcHDZ* genes’ transcripts in leaves of seedlings 2, 4 and 7 d after drought stress and 2 h, 2 d and 7 d after salinity stress based on RNA-seq data. As shown in Fig. 6, expression levels of 15 *JcHDZ* genes were at least two-fold higher or lower, relative to controls, during responses to at least one stress at least at one time point. Of these 15 differentially expressed genes, nine (*JcHDZ06*, *07*, *09*, *16*, *17*, *21*, *25*, *27* and *28*) were significantly up-regulated or down-regulated under both drought and salinity stresses, five (*JcHDZ03*, *04*, *12*, *14* and *22*) only responded to drought stress, and one (*JcHDZ18*) responded only to salinity stress. *JcHDZ16* was significantly down-regulated at all time points in response to salinity stress, and thus was selected for subsequent functional analysis.

### JcHDZ16 is a nucleus-localized transcriptional activator

**Fig. 7:**
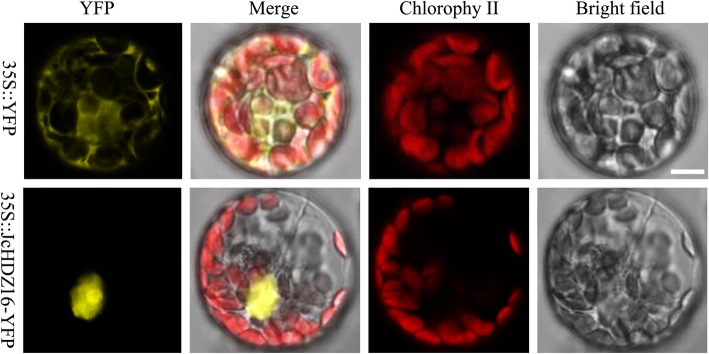
Subcellular localization of *JcHDZ16* gene in *Arabidopsis* protoplasts incubated with 35S::YFP or 35S::JcHDZ16-YFP constructs, as described in *Materials and Methods*. YFP and JcHDZ16-YFP fusion proteins were transiently expressed under control of the CaMV 35S promoter and observed with a laser scanning confocal microscope

To determine the subcellular localization of the protein encoded by *JcHDZ16*, we constructed a 35S::JcHDZ16-YFP fusion vector, which we used to transform *Arabidopsis* protoplasts. We then measured fluorescence signals from the protoplasts (and controls transformed with a 35S::YFP vector) by laser scanning confocal microscopy. As shown in Fig. 7, we observed strong fluorescence signals throughout the whole cells from the control vector, but only in the nuclei of cells harboring the 35S::JcHDZ16-YFP fusion vector. The results clearly suggest that *JcHDZ16* encodes a nuclear protein.

To determine whether *JcHDZ16* had transcriptional activator activity, the full-length cDNA of *JcHDZ16* were cloned into the vector pBD, and then the PEG (polyethylene glycol)-mediated method was used to transfer plasmids from the pBD-JcHDZ16 fusion effector vector and p5 × GAL-Reporter vector into *Arabidopsis* protoplasts. Our data suggested that the ratio of LUC/REN was significantly higher in the experimental group (pBD-JcHDZ16) than in the control group (pBD) (Additional file 4). These results indicate that *JcHDZ16* acts as a transcriptional activator.

### JcHDZ16 binds to the H-box motif

To investigate the binding of JcHDZ16 to the H-box motif, three tandem copies of H-box motif sequence were cloned into pHIS2 and their interactions with JcHDZ16 were determined using Y1H analysis. The results indicate that yeast cells cotransformed with JcHDZ6-effector and different reporters grew on the SD/−Trp/−His/−Leu/ containing 50 mM 3-AT (3-amino-1, 2, 4-triazole) medium, demonstrating that JcHDZ16 can bind to the H-box motif (Additional file 5).

### Phenotype analysis and generation of transgenic rice with *JcHDZ16*

**Fig. 8:**
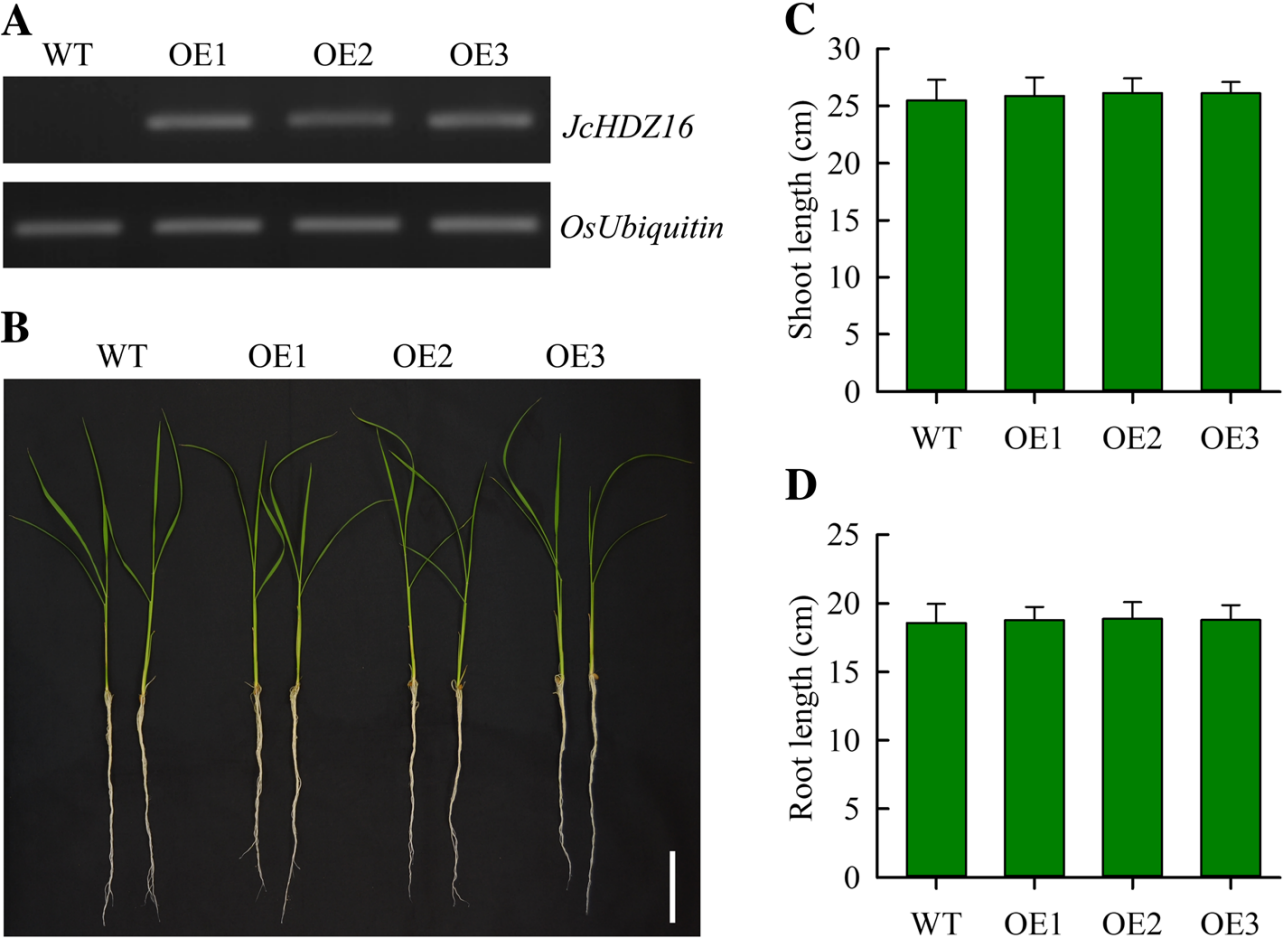
Results of expression and phenotypic analysis in wild-type and transgenic (OE1, OE2, and OE3) rice plants expressing *JcHDZ16*, after 2 weeks growth on Yoshida’s culture solution. **a** Results of RT-PCR analysis. **b** Images of representative seedlings, bar = 6 cm. **c**, **d** Shoot and lengths of the transgenic and wild-type plants: means of *n* = 30 ± SD from three independent biological replicates

To determine the *JcHDZ16* gene’s function in plants, and assess the feasibility of using *JcHDZ* genes to manipulate stress responses in an important crop plant, we overexpressed it in rice. Transformed (*OeJcHDZ16*) rice lines were selected using hygromycin, and RT-PCR analysis confirmed that *JcHDZ16* was expressed in *OeJcHDZ16* lines but not in wild-type lines (Fig. 8a). T3 homozygous lines were used for the following experiments. Phenotypic analysis showed that growth of *JcHDZ16* transgenic plants was similar to that of wild-type plants, and there were no significant differences in root and shoot lengths between them, under non-stressed conditions (Fig. 8b-d). Thus, *JcHDZ16* expression has little apparent effect on rice plants’ growth and development in the absence of stress.

### *JcHDZ16* negatively regulates salinity-induced responses in rice

**Fig. 9:**
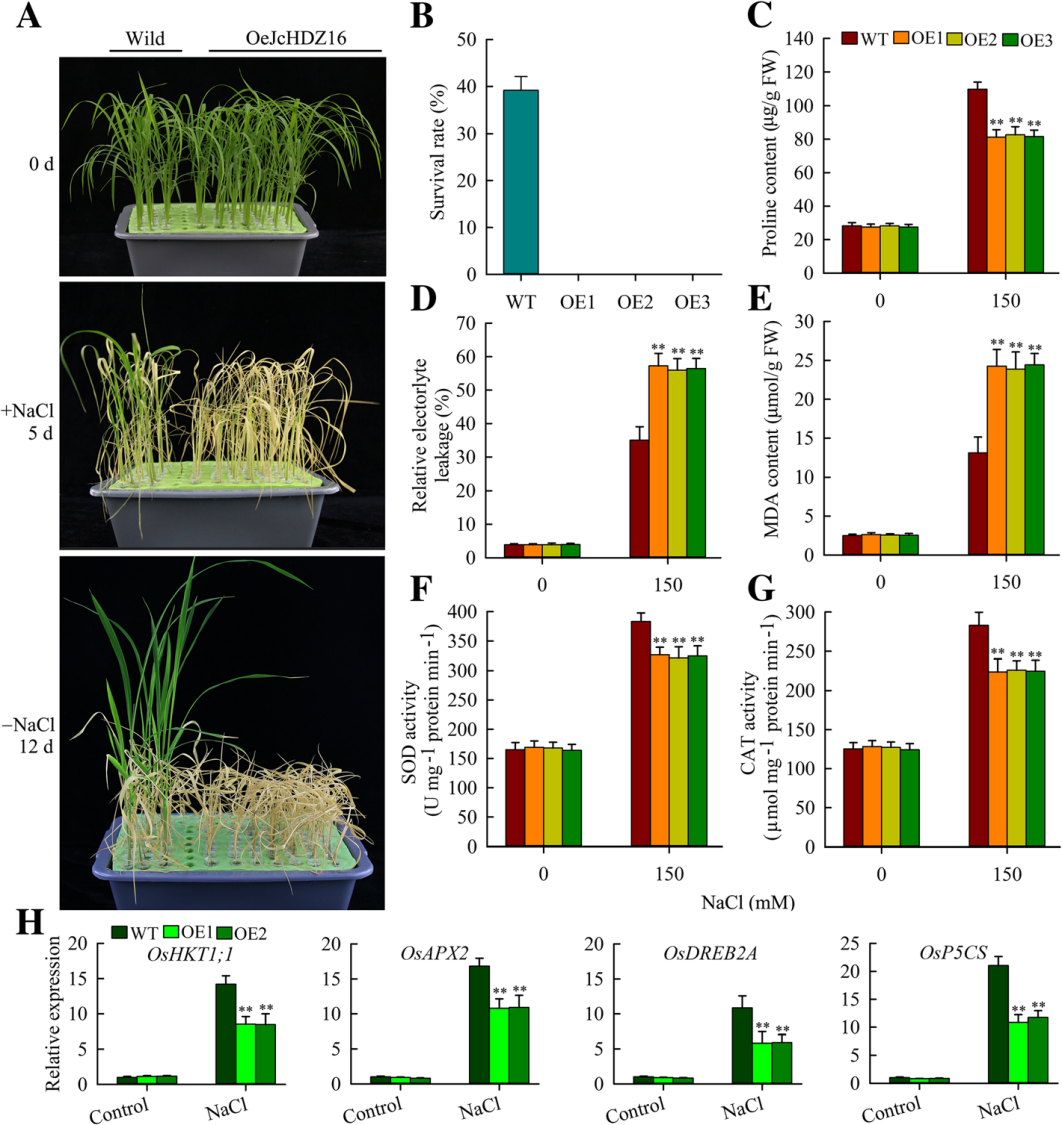
Results of salinity tolerance analysis of wild-type and transgenic rice plants expressing *JcHDZ16*. **a** Wild-type and transgenic plants before and after exposure to 150 mM NaCl, and after a 12-day recovery period (representative images of plants in an experiment with three biological replicates). **b** Survival rates of wild-type and transgenic plants after the 12-day period. **c**-**g** Proline contents, relative electrolyte leakage (REL), MDA contents and activities of catalase (CAT) and (**g**) superoxide dismutase (SOD) in leaves before and after salt treatment. Data in **c**-**g**: means of *n* = 20 ± SD from three independent experiments, asterisks above the bars indicate significant differences from wild-type controls at *p* < 0.01 (**c**-**e**) or *p* < 0.05 (**f**, **g**). **h** Relative expression levels of salt stress-responsive genes, in an experiment with three biological replicates, each with two technical replicates (means of *n* = 6 ± SD, asterisks above the bars indicate significant differences from wild-type controls at *p* < 0.01)

As mentioned above, *JcHDZ16* expression was significantly down-regulated under salinity stress, indicating that it may play a negative role in regulation of salinity responses. To test this hypothesis, we further examined effects of *JcHDZ16* overexpression on salinity tolerance in rice, as follows. Two-week-old *OeJcHDZ16* and wild-type rice seedlings were exposed to Yoshida’s culture solution supplemented with 150 mM NaCl for 5 days. After this treatment, leaves of all tested *OeJcHDZ16* seedlings showed clear signs of damage (severe rolling and yellowing), while 65.9% of the wild-type seedlings rice remained green (Fig. 9a). After a 12-day recovery period under normal (non-stressed) conditions, all the *OeJcHDZ16* seedlings were dead, whereas approximately 39% of the wild-type seedlings survived and grew again (Fig. 9b).

Proline accumulation has been shown to be an important adaptation mechanism in plants’ responses to salinity and drought stresses [35]. We found no significant difference in proline content between *OeJcHDZ16* and wild-type lines under normal conditions, but it was clearly higher in wild-type lines under salinity stress (Fig. 9c), clearly indicating that this stress response is repressed in the *OeJcHDZ16* lines. In addition, relative electrolyte leakage (REL) and MDA levels (indicators of cell membrane damage) of *OeJcHDZ16* leaves were higher than those of wild-type plants under the salinity stress (Fig. 9d and e). These results suggest that leaf cells are damaged less by salinity in wild-type plants than in the *OeJcHDZ16* plants. In further tests, we found that catalase (CAT) and superoxide dismutase (SOD) activities were significantly higher in wild-type leaves than in *OeJcHDZ16* leaves under salinity stress, but not under normal growth conditions (Fig. 9f and g). Taken together, these data strongly confirm that transgenic expression of *JcHDZ16* can reduce rice plants’ salinity tolerance.

Overexpression of salinity stress-related genes (including *OsHKT1;1*, *OsAPX2*, *OsDREB2A*, *OsP5CS* or their homologs) can increase plants’ rice salinity tolerance [36–39]. Therefore, to clarify *JcHDZ16*’s role in regulation of salinity responses in the transgenic rice lines, we estimated (by qRT-PCR) relative transcript levels of these genes in leaves of wild-type and *OeJcHDZ16* plants under normal and salinity stress conditions. The results revealed that their expression levels were upregulated in leaves of *OeJcHDZ16* plants in response to salinity stress, but less strongly than in wild-type (Fig. 9h). In contrast, no clear differences in their expression levels between the wild-type and *OeJcHDZ16* plants were detected under normal growth conditions (Fig. 9h).

### Transgenic expression of *JcHDZ16* increases ABA sensitivity in rice

**Fig. 10:**
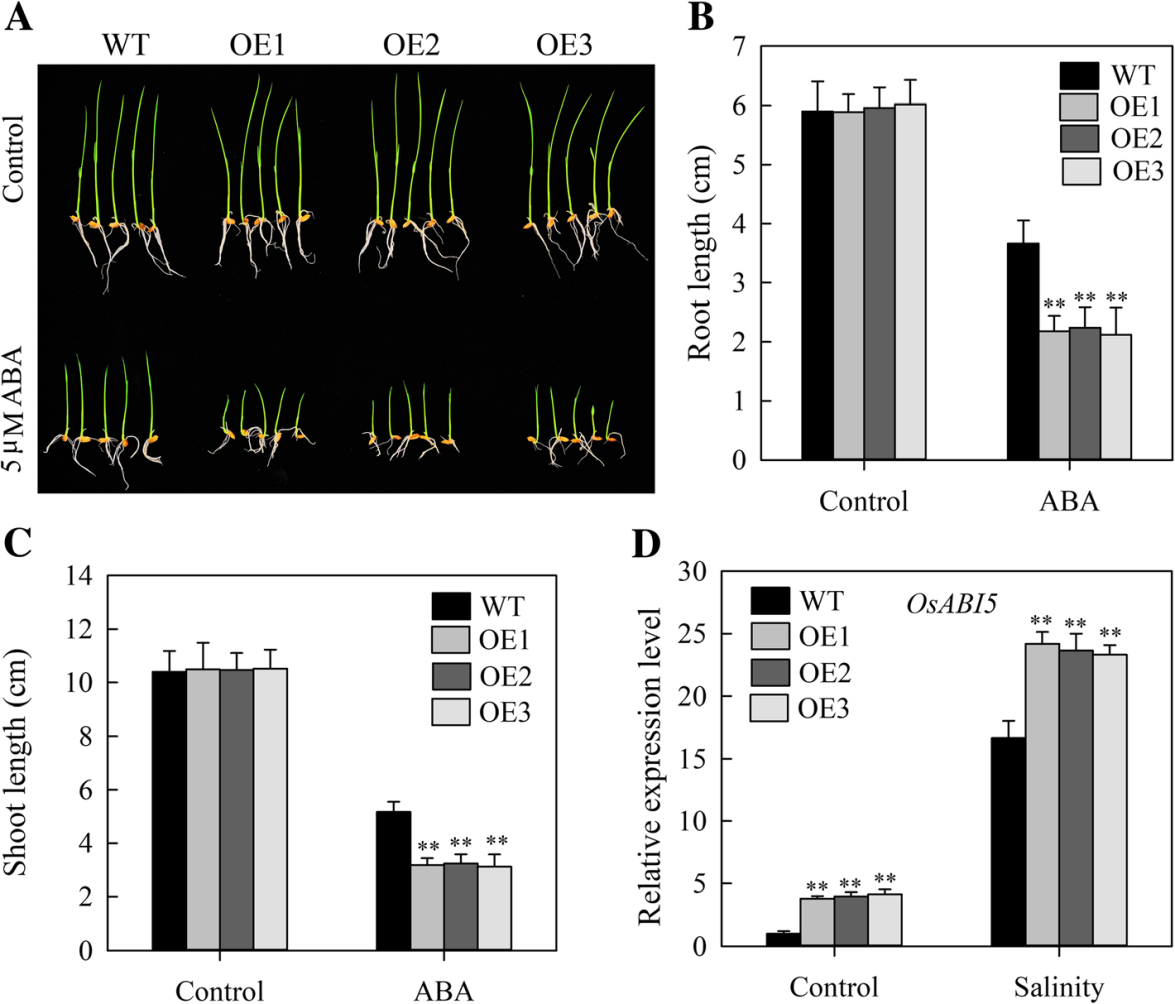
Results of ABA-sensitivity assay of *JcHDZ16*-expressing transgenic rice plants. **a** Phenotypes of wild-type and transgenic plants grown on Yoshida’s culture solution with 5 μM ABA. **b** Root lengths of transgenic and wild-type plants after 4 days growth on Yoshida’s culture solution with and without 5 μM ABA (means of *n* = 30 ± SD from three independent biological experiments, asterisks above the bars indicate significant differences from wild-type controls at *p* < 0.01). **c** Shoot lengths of transgenic and wild-type plants after 4 days growth on Yoshida’s culture solution with and without 5 μM ABA (means of *n* = 30 ± SD from three independent biological experiments, asterisks above the bars indicate significant differences from wild-type controls at *p* < 0.01). **d** Relative expression levels of the *OsABI5* gene, in an experiment with three biological replicates, each with two technical replicates (means of *n* = 6 ± SD, asterisks above the bars indicate significant differences from wild-type controls at *p* < 0.01)

As ABA plays important roles in responses to abiotic stress [1], we also examined effects of transgenic *JcHDZ16* expression on plants’ sensitivity to exogenous ABA, by exposing rice seedlings to Yoshida’s culture solution with and without 5 μM ABA (Fig. 10a). Four days later, all tested transgenic lines had similar shoot and root lengths to wild-type controls under normal growth conditions, but significantly shorter shoots and roots than wild-type plants under the ABA treatment (Fig. 10b and c). Thus, transgenic expression of *JcHDZ16* increased their sensitivity to ABA. We also found that *OsABI5* expression was higher in transgenic than in wild-type plants under both normal growth and salt stress conditions (Fig. 10d). Taken together, these results strongly suggest that *JcHDZ16* acts as a negative regulator in responses to salt stress through ABA-mediated signal transduction pathways.

## Discussion

Physic nut is being widely cultivated, partly because of its high drought and salinity tolerance [31, 32]. In model plants such as *Arabidopsis* and rice, significant progress has been made in elucidating functions of abiotic stress-related genes [40, 41]. Results have shown that HD-ZIP proteins are some of the most important transcription factors involved in abiotic stress signaling pathways [15, 16], and group I genes of the family strongly contribute to tolerance of abiotic stresses, including drought and salinity [29, 30]. However, there is little knowledge of the molecular mechanisms involved in physic nut’s stress tolerance generally, or the identities, expression profiles and functions of its HD-ZIP genes. Therefore, we identified, characterized and examined expression profiles of *HD-ZIP* genes in the species, and selected one (designated *JcHDZ16*) that was clearly repressed under salinity stress, and further analyzed its function by transgenically expressing it in rice.

In total, 32 *JcHDZ* genes were identified in the physic nut genome (Additional file 1) of 320 Mb [33], fewer than reportedly present in the 466 Mb rice genome (44), 125 Mb *Arabidopsis* genome (48), and 2300 Mb maize genome (55) [11, 15, 16]. This may be at least partly because *HD-ZIP* genes in the physic nut genome were not subjected to segmental and tandem duplication events during the species’ early evolutionary history [33], whereas such duplications made major contributions to the expansion of *HD-ZIP* genes in rice, *Arabidopsis* and maize [11, 15, 16]. Following the classification of *HD-ZIP* genes from rice, *Arabidopsis* and maize [11, 15, 16], members of the physic nut HD-ZIP family were divided into four groups, and group III had the fewest members (Fig. 1), as in other plants such as *Arabidopsis*, rice, maize, wheat, poplar, grape, soybean and cassava [11, 13–19]. These results show that the HD-ZIP III group is highly conserved in physic nut and other plants, confirming previous studies [42].

The extent of differences in exon-intron patterns among plant species is closely related to their evolution. We found that the exon-intron splicing arrangements and numbers of exons of *JcHDZ* genes were similar to those reportedly observed in *HD-ZIP* genes from rice, maize and *Arabidopsis* [11, 15, 16]. For example, members of group I appear to have 1 to 3 exons in physic nut (our findings, Fig. 2), *Arabidopsis*, maize and rice [11, 15, 16]. Furthermore, most JcHDZ proteins of the same group apparently have similar exon-intron structures (Fig. 2). The structural similarities of members of the same group support the classification of HD-ZIP proteins in physic nut, and the conserved motifs provide further support. For example, we found that group III and IV genes have more motifs than genes of groups I and II in physic nut, motif 14 was absent in group I and a MEKHLA domain was only detected in group III (Fig. 3). Similar patterns have also been detected in various dicot and monocot plants, for example poplar, grape, rice, wheat, soybean, maize, *Arabidopsis* and cassava [11, 13–19]. Taken together, the similarities in gene structure and conserved motifs of *JcHDZ* genes in the same group corroborate their classification and inferred evolutionary relationships.

Gene expression profiles can provide valuable indications of genes’ biological functions, so we explored those of the 32 *JcHDZ* genes using RNA-seq data (Fig. 5). The results suggest that *JcHDZ10* is most highly expressed in seeds. Moreover, its homologs in *Arabidopsis AtML1* and *PDF2* are essential for embryo survival [43], indicating that *JcHDZ10* may be involved in physic nut seed development. *PHB*, another HD-ZIP gene, is involved in regulating root length by directly activating the cytokinin biosynthesis gene *IPT7* [44], and its homolog in physic nut *JcHDZ17* was most strongly expressed in roots. Thus, we hypothesize that it may play a vital role in root growth and development by regulating expression of cytokinin biosynthesis genes. Similarly, *JcHDZ25* was highly expressed in roots, and its *Arabidopsis* homolog *AtHB12* induces root elongation in young transgenic plants [45], suggesting that *JcHDZ25* may also be involved in regulating root elongation. High levels of *JcHDZ01* transcripts were detected in stem cortex, and its *Arabidopsis* homolog *HB52* is also highly expressed in stems [46], suggesting that *JcHDZ01* may participate in stem development. The *Picea glauca* gene *PgHZ1* is mainly expressed in embryos, and its products are apparently required for embryonic growth, according to experiments with transgenic *Arabidopsis* [47]. Its homolog in physic nut, *JcHDZ06*, is preferentially expressed in seeds, suggesting that *JcHDZ06* may have a similar function to the *Picea glauca* gene in the course of plant growth and developmental. *JcHDZ14*, *16*, *22* and *28* had constitutive expression patterns in tested organs, indicating that they probably participate in fundamental elements of plant growth and development processes. Taken together, we infer that *JcHDZ* genes act in diverse aspects of developmental processes in physic nut, and further study is required to elucidate their roles.

Whole-genome expression analyses have demonstrated that abiotic stresses induce reductions in expression of some *HD-ZIP* genes in various plants [18]. For example, 59 *HD-ZIP* genes are reportedly involved in responses to salinity or drought stress in soybean [13], 16 are up-regulated or down-regulated under salinity or drought stress in poplar [19], and 28 are regulated by drought or salinity in wheat [14]. In addition, overexpression or knock-out of several *HD-ZIP* genes has demonstrated capacity to increase plants’ tolerance to drought and salinity stresses [29, 30, 42]. For example, expression of *TaHDZipI-5* increases drought tolerance of wheat [42], and transgenic *Zmhdz10*-expressing rice and *Arabidopsis* plants display increased tolerance of salinity and drought [30]. Previous studies have clearly shown that physic nut has high drought and salinity resistance [31, 32]. However, no information on responses of *HD-ZIP* genes to salinity and drought in physic nut has been previously published. In this study, transcriptome sequencing data generated from physic nut exposed to drought and salinity enabled us to identify 15 *JcHDZ* genes that are apparently involved in responses to these stresses (Fig. 6). For example, expression of *JcHDZ06*, *07*, *09*, *16* and *28* was up- or down-regulated during both the drought and salinity treatments at one time point at least, whereas *JcHDZ03* only responded to drought stress. Furthermore, numerous investigations have shown that *HD-ZIP* genes of group I participate in regulation of abiotic stresses responses [29, 30]. Similarly, we found that expression of most *JcHDZ* genes of group I increased or decreased during the drought and/or salinity treatments (Fig. 6). Collectively, our results suggest these *JcHDZ* genes may play important roles in regulating plant responses to drought and salinity stress, and their specific functions need to be determined by transgenic analysis.

Salinity is a major inhibitor of plant growth and development that severely impairs crop yields in many regions [48], thus there are urgent needs to elucidate salinity tolerance mechanisms and identify ways to counter the impairments. We found that *JcHDZ16*, a gene of group I, was clearly down-regulated in leaves of physic nut under salinity stress (Fig. 6), and to explore its function we tested its transgenic effects. For this we used rice, although *JcHDZ* genes were closer to homologs in *Arabidopsis* than to rice homologs. However, the degree of homology was high in both cases. Thus, we took the opportunity to test the feasibility of using *JcHDZ* genes to modify the stress resistance of an important crop plant in addition to verifying its functional role. Transgenic expression of *JcHDZ16* increased rice plants’ sensitivity to salinity stress, as leaf rolling and loss of chlorophyll were more pronounced in *OeJcHDZ16* plants than in wild-type controls (Fig. 9).

When plants are exposed to environmental stresses such as drought and salinity, some rapid adaptive physiological responses mediated by osmotic changes may provide rapid and accurate indications of their tolerance of the stresses [49, 50]. Notably, proline plays a major role in counteracting effects of drought and salinity stresses, as an osmoprotectant or ‘compatible solute’ in diverse species [35]. A close positive relationship between capacity to accumulate proline and stress tolerance has been confirmed by overexpressing and knocking out the *P5CS* (proline synthesis) gene in various plants [36, 51, 52]. We found that leaves of wild-type plants accumulate more proline than leaves of *OeJcHDZ16* plants under salinity stress (Fig. 9c), which may account for at least some of the *OeJcHDZ16* plants’ higher sensitive to salinity stress. In addition, salinity induced higher increases in REL and MDA contents in leaves of *OeJcHDZ16* plants than in leaves of wild-type plants (Fig. 9d and e). These findings indicate that salinity causes more cell membrane damage in leaves of *OeJcHDZ16* plants, corroborating *JcHDZ16*’s role in negative regulation of salinity responses.

CAT and SOD also play key roles in plants’ tolerance of abiotic stresses (including drought and salinity) by scavenging excess reactive oxygen species (ROS) [53, 54]. For example, transgenic *ADC2* expression enhances *Arabidopsis* plants’ tolerance of salt stress by increasing CAT and SOD activities [55]. We found that *OeJcHDZ16* plants had lower CAT and SOD activities under salinity stress than wild-type plants (Fig. 9f and g), indicating that salinity may induce more severe oxidative damage in them than in wild-type plants. Collectively, these results at least partially explain the increased sensitivity to salinity of our transgenic rice plants.

In addition to physiological factors, various salt stress-related genes have been identified [40, 41, 56]. For example, overexpression of *JcERF11* increases sensitivity to salt stress in rice by reducing expression of *HKT1;1*, *HKT1;5*; *APX2* and *SNAC1* [8]. As in previous studies, we observed that expression levels of some salt-stress-related genes (*OsHKT1;1*, *OsAPX2*, *OsDREB2A* and *OsP5CS*) were significantly lower in the *OeJcHDZ16* plants than in wild-type controls in response to salinity stress (Fig. 9h). *OsHKT1;1* participates in removal of Na^+^ from leaf blades [39], *OsAPX2* gene products are involved in scavenging ROS [38], and overexpression of *OsP5CS* or *OsDREB2A* increases rice plants’ accumulation of proline under salt stress [36, 37]. Transgenic expression of these genes also enhances plants’ salt tolerance [36–39]*.* Thus, our results strongly indicate that *JcHDZ16* negatively regulates salinity responses in our transgenic rice lines at least partly through down-regulation of known salt stress-related genes. Since reporter gene studies in *Arabidopsis* protoplasts suggested that *JcHDZ16* acts as a transcriptional activator, its function as a negative regulator in stress (salinity) responses might be explained via activation of other repressors.

Previous studies have shown that ABA signaling plays essential roles in plant responses to abiotic stresses, including drought and salinity, and stress-responsive genes generally participate in either ABA-dependent or ABA-independent pathways [1]. Our results clearly indicate that *JcHDZ16*-expressing rice seedlings were more sensitive to ABA than wild-type seedlings (Fig. 10a). Other HD-ZIP proteins, such as *Zmhdz10* and *Oshox22*, reportedly have similar effects [30, 56]. In addition, we found that *OsABI5* expression was significantly higher in our transgenic plants than in the wild-type plants (Fig. 10d). *OsABI5* is an important regulator in the ABA signaling pathway, and it overexpression both increases rice plants’ sensitivity to ABA and reduces their salt tolerance [57]. Thus, *JcHDZ16* may be involved in ABA signal transduction and the associated increased salt sensitivity may be partly due to up-regulated expression of *OsABI5*. In summary, the data from the present study strongly indicate the important functions of *JcHDZ16* in response to salt stress through the ABA signal transduction pathway.

## Conclusion

We have identified 32 full-length *JcHDZ* genes, which can be robustly assigned to four phylogenetic groups, according to their exon-intron structures and conserved motifs. Their expression profiles clearly indicate that some *JcHDZ* genes are involved in responses to abiotic stresses. Transgenic expression of one of the genes (*JcHDZ16*) reduced the tolerance of rice plants to salinity stress, corroborating the hypothesis that some of these genes participate in physic nut’s responses to abiotic stresses. The transgenic seedlings were also more sensitive to ABA. Thus, *JcHDZ16* appears to be a negative regulator of salt stress responses acting through ABA signaling pathways. In summary, our results identify candidate genes for future functional analysis of *JcHDZ* genes involved in salt-related signaling pathways. They also provide indications of the phylogeny, structural features, and functions of *HD-ZIP* genes in physic nut, but much further analysis is required.

## Methods

### Plant materials and growth conditions

The inbred cultivar GZQX0401 of *J. curcas* was used for our research, since its genome has been fully sequenced [33]. Seeds of the cultivar were obtained from South China Botanical Garden, Chinese Academy of Sciences, Guangzhou, China. The wild-type rice (*Oryza sativa* L.) cultivar used was the japonica cv. Zhonghua 11 (ZH11). Seeds were germinated and cultured in soil in basins in a greenhouse under natural sunlight at Zhoukou Normal University, China.

### Identification of *JcHDZ* genes in physic nut

To identify putative physic nut HD-ZIP proteins, HMM models of the two characteristic domains of a HD-ZIP transcription factor, homeobox associated leucine zipper (LZ, PF02183) and homeobox (HD, PF00046), were downloaded from PFam (http://pfam.sanger.ac.uk/). They were then used as query sequences in local HMM-based searches, setting E-values < 0.01 [16]. In addition, to identify physic nut HD-ZIP proteins that might have been missed through HMM model searching, BLASTP searches against the physic nut genomic databases (available from DDBJ/EMBL/GenBank under accession number AFEW00000000) were performed using all HD-ZIP gene family members in *Arabidopsis* and rice as query sequences. PFam and SMART (http://smart.embl-heidelberg.de/) databases were used to examine all candidate HD-ZIP protein sequences. Then, to further confirm the conserved domains of predicted HD-ZIP proteins from physic nut, multiple sequence alignments were performed using Clustal X software. ExPASy (https://web.expasy.org/protparam/) was used to analyze the identified JcHDZ proteins’ physical and chemical characteristics.

### Phylogenetic and gene structure analysis

HD-ZIP protein sequences of *Arabidopsis* were downloaded from the *Arabidopsis* Information Resource (TAIR, https://www.arabidopsis.org/, Additional file 5). HD-ZIP proteins of rice and maize were downloaded from the Phytozome (http://www.phytozome.net/) and NCBI (https://www.ncbi.nlm.nih.gov/) websites (Additional file 6), and physic nut sequences were downloaded from GenBank (http://www.ncbi.nlm.nih.gov/; available from DDBJ/EMBL/GenBank under accession number AFEW00000000). Following sequence alignment of HD-ZIP proteins from all these plants by ClustalX, MEGA 6 was used to construct a Neighbor-Joining tree by bootstrapping with the following parameters: 1000 bootstrap replications, Poisson model, and treatment of gaps/missing data as complete deletions. In addition, CDS and genomic sequences were submitted to GSDS (Gene Structure Display Server, http://gsds.cbi.pku.edu.cn/) to obtain schematic diagrams of the genes’ structures.

### Conserved motifs and chromosomal localization

Conserved motifs of the JcHDZ proteins were analyzed using MEME software (http://meme-suite.org/) with the following parameters: motif site distribution, zero or one site per sequence; motif count, 15; and motif width, 6–100. Chromosomal locations of *JcHDZ* genes were obtained from previously published information [33], using a maximum likelihood mapping algorithm and the Kosambi mapping function to calculate map distances in cM [33], and linkage maps for the *JcHDZ* genes were drawn using the MapChart software package.

### Expression profile analysis of *JcHDZ* genes

Physic nut seedlings were grown in the big round basin containing soil (30%) and sand (70%) at 30 °C in natural sunlight in a temperature-controlled greenhouse. After emergence of the first true leaf, the seedlings were irrigated with Hoagland nutrient solution (volume 1 L, pH 6.0) once every 2 days at eight o’clock in the morning. Roots, stem cortex, and leaves of seedlings were collected at the six-leaf stage. In addition, seeds were collected at both an early developmental stage at 14 DAP (designated S1) and the filling stage at 41 DAP (designated S2). The sampled materials were immediately stored at − 80 °C until required for gene expression analysis (Additional file 3). For the drought stress treatment, we stopped watering seedlings at the six-leaf stage, and collected leaves 2, 4 and 7 d after the treatment commenced. For the salinity stress treatment, Hoagland nutrient solution containing 100 mM NaCl was used to irrigate seedlings from the six-leaf stage every day at eight o’clock in the morning, and we collected leaves 2 h, 2 d and 4 d after the treatment commenced. The sampled leaves were stored immediately at − 80 °C until required for gene expression analysis, when raw sequence data were acquired following standard protocols [8] and submitted to the sequence read archive (SRA) at NCBI (with accession nos. PRJNA244896 and PRJNA257901 for the salinity stress and drought stress data, respectively).

### Subcellular localization

The open reading frame (ORF) sequence of the *JcHDZ16* gene without the stop codon was amplified by RT-PCR using using cDNA generated from RNA extracted from physic nut root and leaf samples as a template. After confirmatory DNA sequencing, the sequence was connected to the pSAT6-eYFP-N1 vector, then a pSAT6-JcHDZ16-YFP fusion expression vector, with a CaMV 35S promoter (35S), was established. For subcellular localization of JcHDZ16 protein, a 35S::YFP vector was used as a control, and the PEG (polyethylene glycol)-mediated method was used to transfer plasmids from the 35S::JcHDZ16-YFP fusion expression vector and control vector into *Arabidopsis* protoplasts. The transformed protoplasts cells were incubated at 25 °C for 16 h, then laser scanning confocal microscopy was used to detect YFP fluorescence signals. *Arabidopsis* protoplasts were prepared following Axelos [58].

### Transactivation assay

The full-length cDNA of *JcHDZ16* amplified by PCR using specific primers was inserted into the *Kpn* I and *Xba* I sites to creat a fusion construct of pBD-JcHDZ16. The pBD-JcHDZ16 plasmid and p5 × GAL-Reporter plasmid were transformed into *Arabidopsis* protoplasts. An empty pBD vector was used as a negative control. Total protein from *Arabidopsis* protoplasts was extracted using ProteoPrep® Total Extraction Sample Kit (Sigma) based on the manufacturer’s instructions, and then the enzyme-labeled instrument (TECAN LAI-2000) was used to analyze the fluorescent activity of proteins. Transcriptional activation was analyzed according to the ratio of LUC/REN.

### Examining the binding of *JcHDZ16* to the H-box motif using Y1H

Three tandem copies of H-box were inserted into pHIS2 (Clontech) upstream of the reporter gene HIS3. The CDS of *JcHDZ16* was cloned into pGADT7-Rec2 (Clontech) as the effector (pGADT7-JcHDZ16). The constructs were cotransformed into Y187 cells, which were plated onto SD/−Trp/−His/ and SD/−Trp/−His/−Leu/ medium supplemented with 50 mM 3-AT (3-amino-1, 2, 4-triazole) and incubated at 30 °C for 3–5 days.

### Gene cloning and plant transformation

To construct a *JcHDZ16* overexpression vector, cDNA containing its full-length CDS was amplified by RT-PCR from total RNA isolated from physic nut leaf and root samples using primers shown in Additional file 4. The PCR product was cloned into the pMD18-T vector (Takara, http://www.takara.com.cn/), and successful amplification of the target gene was confirmed by DNA sequencing. *Kpn* I and *Xba* I were used to excise the target sequence from the pMD18-T vector, then the CDS was cloned into the pCAMBIA1301 vector at the *Kpn* I/*Xba* I site under control of the 35S promoter and ocs (octopine synthase) terminator.

The resulting construct was transformed into *Agrobacterium* (strain EHA105) by the freeze–thaw procedure, then *Agrobacterium* lines harboring the constructs were used to transform and regenerate rice seedlings, following published protocols [8]. Transgenic plants were confirmed through hygromycin screening and semi-quantitative reverse transcription PCR analysis. T3 homozygous lines were used for subsequent experiments.

### Stress treatments

For salinity stress treatment, 2-week-old rice seedlings in a growth chamber providing 16 h light/8 h dark cycles at 25 °C were transferred to Yoshida’s culture solution containing 150 mM NaCl for 5 days, returned to Yoshida’s culture solution for 12 days, then survival rates were calculated. In addition, leaves from two-week-old rice seedlings exposed to this salinity stress treatment for 2 days were used for qRT-PCR analysis. For ABA sensitivity assays, approximately 0.5 cm tall rice seedlings were exposed to Yoshida’s culture solution supplemented with 5 μM ABA (or without supplements, for controls) for 4 days, then their shoot lengths were measured. Similar results were obtained with three biological replicates.

### Measurements of physiological parameters

Leaves from two-week-old rice seedlings exposed to salinity stress for 2 days were used to analyze relative electrolytic leakage (REL), proline and malondialdehyde (MDA) contents, and activities of CAT and SOD. For REL measurements, about 0.2 g leaf samples were washed five times with deionized water, then placed in test tubes, followed by 10 mL of deionized water. Each sample was vibrated continuously at 25 °C for 2 h, then the conductivity (C1) of the solution was measured using a top conductivity meter. Next, each sample was boiled for 20 min, the resulting solution was cooled to room temperature, the conductivity (C2) was measured again and REL was simply calculated from REL (%) = C1/C2 × 100. Previously described methods were used to estimate samples’ proline contents [59], MDA contents [60], and activities of CAT and SOD activities in leaves from wild-type and transgenic rice [61].

### RNA isolation and qRT-PCR analysis

To estimate *JcHDZ16* expression levels in wild type and transgenic rice, total RNA was isolated from selected organs of 2-week-old seedlings that had been sampled and stored at − 80 °C, using a MiniBEST plant RNA extraction kit (TaKaRa Code No. 9769). 2 μg RNA samples were used to synthesize first-strand cDNA using M-MLV reverse transcriptase (Promega, http://www.promega.com). *JcHDZ16* sequences were then quantified by qRT-PCR using SYBR GreenSYBR® Premix Ex Taq™ (TaKaRa, Japan) and a LightCycler® 480 real-time PCR system (http://www.roche.com/), with the following settings: 95 °C for 30 s, followed by 40 cycles at 95 °C for 5 s, 60 °C for 20 s, and 72 °C for 20 s. The cited manufacturers’ instructions were followed in all of these procedures. Relative expression levels were calculated using the 2^-∆∆CT^ method, and *JcActin* and *Osubiquition* as reference genes for physic nut and rice, respectively. All primers used in this study are listed in Additional file 7. Three biological replicates and two technical replicates of each biological replicate were used in this experiment.

### Statistical analysis

Three biological replicates were used for all experiments, and Duncan tests were used to assess the significance of differences in measured variables between the materials [62] with the SAS software package version 9.

## Additional files

Additional file 1: Summary of *JcHDZ* genes encoding HD-ZIP proteins in physic nut. (XLSX 12 kb)

Additional file 2: Motifs in JcHDZ proteins, the amino acid composition of each conserved motif. (TIF 11044 kb)

Additional file 3: Expression levels of the 32 *JcHDZ* genes in tested organs (root, stem cortex, leaf, and seeds) based on RNA-seq data. (XLSX 13 kb)

Additional file 4: Transcriptional activity of of *JcHDZ16* gene. (A) Schematic structures of the plasmids used in dual-luciferase assay to analyze the transcriptional activity of *JcHDZ16*. (B) Dual-luciferase assay suggested that *JcHDZ16* had transcriptional activity. Each experiment with three biological replicates, each with three technical replicates (means of *n* = 9 ± SD, asterisks above the bars indicate significant differences from controls at *p* < 0.01). (TIF 1409 kb)

Additional file 5: Analyses of JcHDZ16 binding motif. (A) Schematic diagram of the effector and reporter constructs used in Y1H analysis. (B) Analysis of binding of JcHDZ16 to H-box using Y1H. (TIF 744 kb)

Additional file 6: Sequences of proteins encoded by HD-ZIP genes in *Arabidopsis*, rice and maize. (TXT 67 kb)

Additional file 7: Primers used in this study. The Primer Premier 5.0 software package (http://www.premierbiosoft.com/primerdesign/) was used to design all gene-specific primers. (XLSX 10 kb)

## Data Availability

Raw data supporting findings of this study have been submitted to NCBI’s sequence read archive (SRA) (accession nos. for the salinity and drought stress data: PRJNA244896 and PRJNA257901, respectively). Acquired sequences of *JcHDZ* proteins are available from DDBJ/EMBL/GenBank under accession no. AFEW00000000NCBI. Other relevant data obtain during the research are included in this published article and associated supplementary information files.

## Abbreviations

CAT: Catalase
HD: Homeodomain
HD-ZIP: Homeodomain-leucine zipper
LZ: Leucine zipper
MDA: Malondialdehyde
ORF: Open reading frame
REL: Relative electrolytic leakage
SOD: Superoxide dismutase
