# Supplementary figures and images for: Genome-wide identification and expression profile of HD-ZIP genes in physic nut and functional analysis of the JcHDZ16 gene in transgenic rice
Source: BMC Plant Biol. 2019 Jul 8;19:298. doi: 10.1186/s12870-019-1920-x (PMC6615155; doi:10.1186/s12870-019-1920-x)

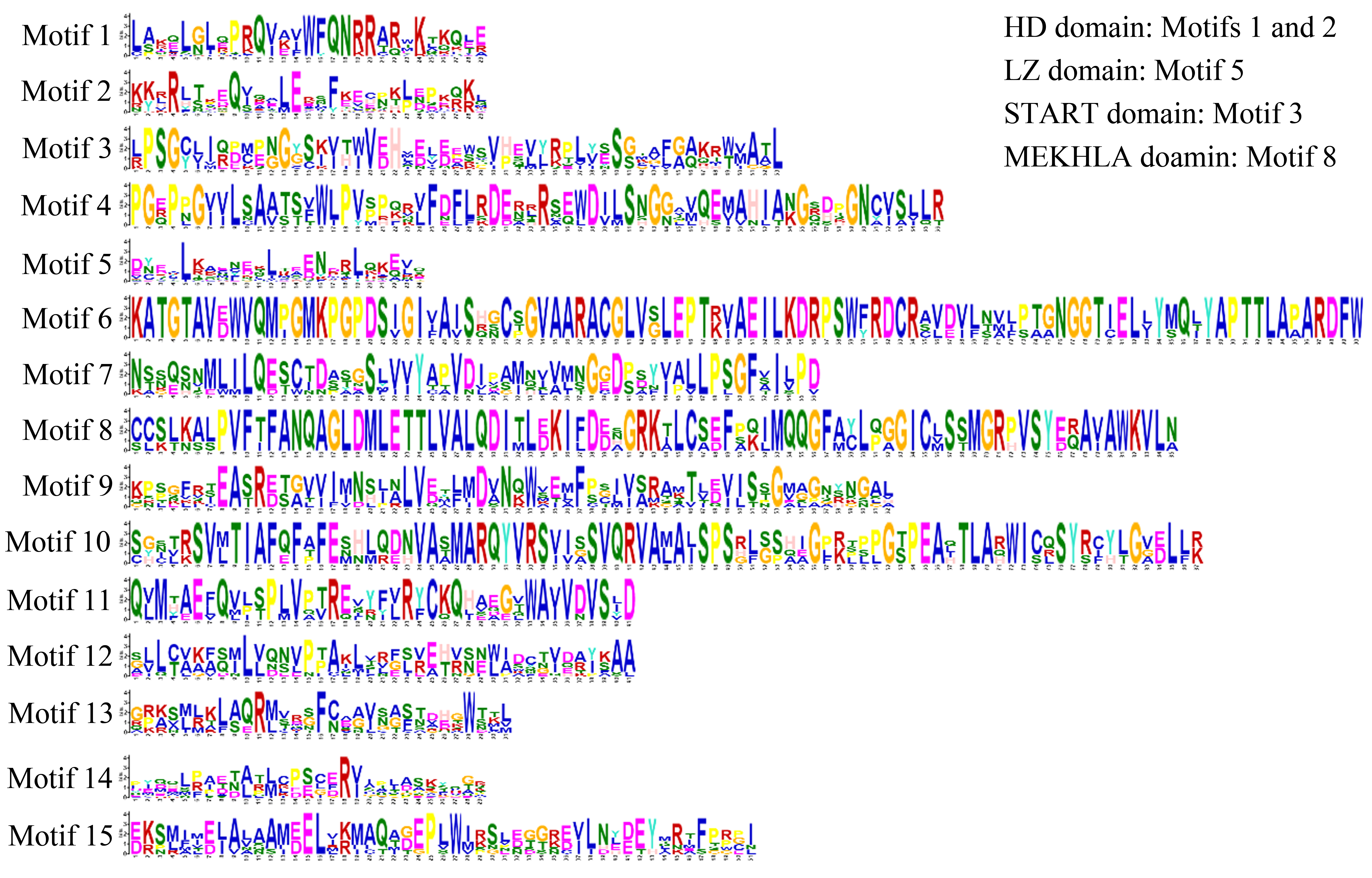

Supplement: Supplementary file 2 — Motifs in JcHDZ proteins, the amino acid composition of each conserved motif. (TIF 11044 kb) [file 12870_2019_1920_MOESM2_ESM.tif]

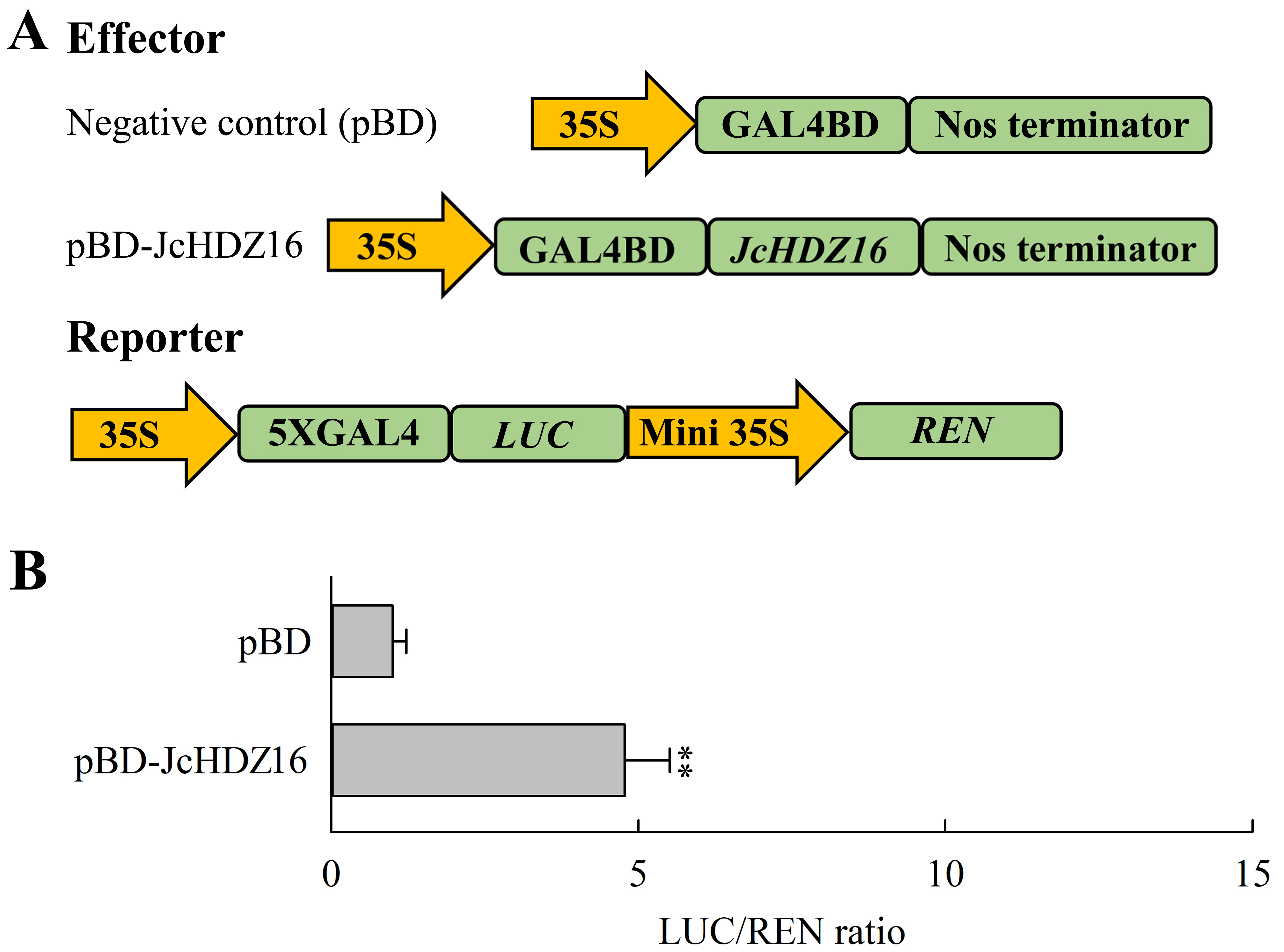

Supplement: Supplementary file 4 — Transcriptional activity of of JcHDZ16 gene. (A) Schematic structures of the plasmids used in dual-luciferase assay to analyze the transcriptional activity of JcHDZ16. (B) Dual-luciferase assay suggested that JcHDZ16 had transcriptional activity. Each experiment with three biological replicates, each with three technical replicates (means of n = 9 ± SD, asterisks above the bars indicate significant differences from controls at p < 0.01). (TIF 1409 kb) [file 12870_2019_1920_MOESM4_ESM.tif]

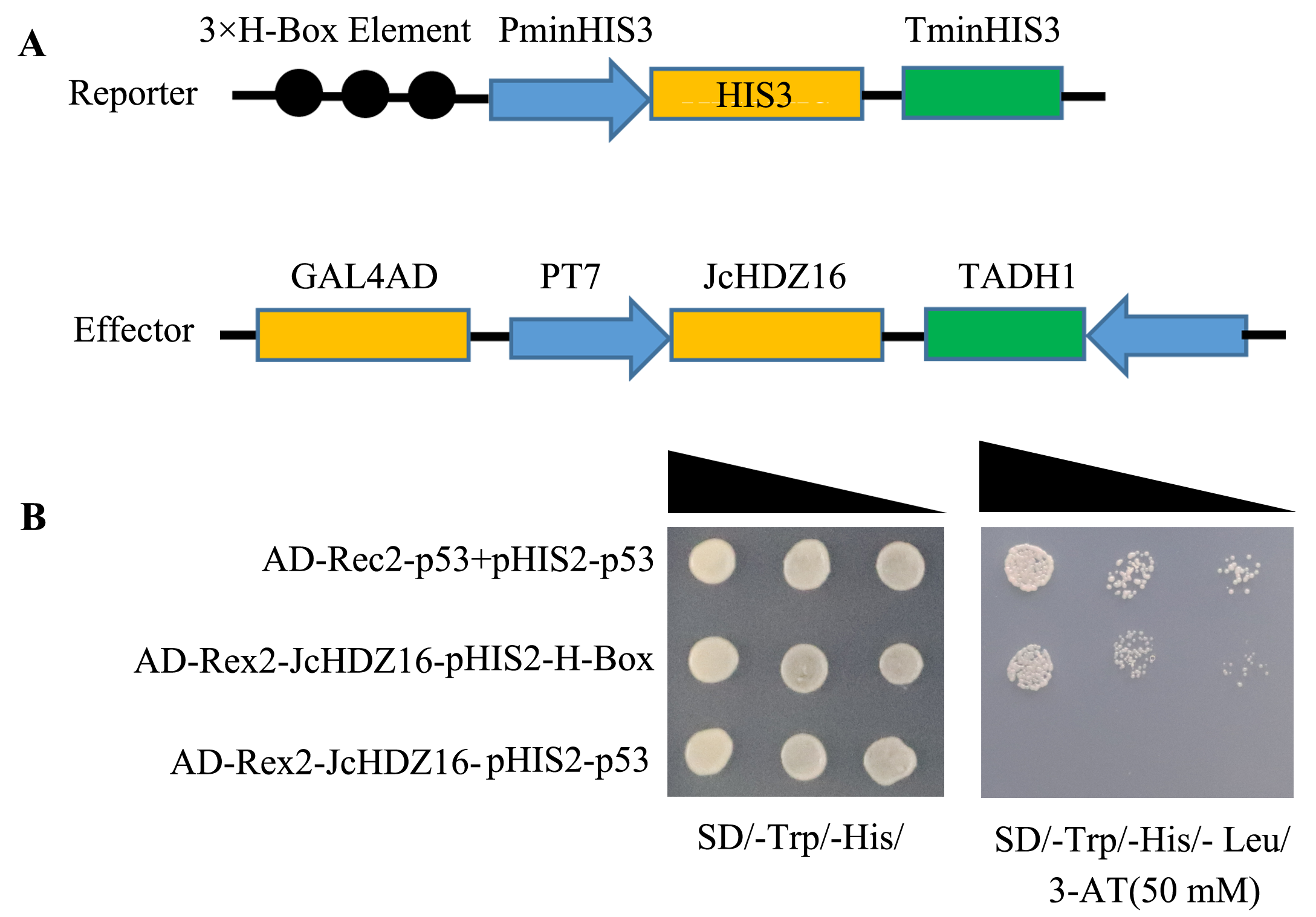

Supplement: Supplementary file 5 — Analyses of JcHDZ16 binding motif. (A) Schematic diagram of the effector and reporter constructs used in Y1H analysis. (B) Analysis of binding of JcHDZ16 to H-box using Y1H. (TIF 744 kb) [file 12870_2019_1920_MOESM5_ESM.tif]
